# Factors Predicting Subjective Satisfaction for Successful Hearing Aid Adaptation

**DOI:** 10.3390/jcm13020398

**Published:** 2024-01-11

**Authors:** Jeong Hun Jang, Jungho Ha, Oak-Sung Choo, Young Sook Kang, Hun Yi Park, Yun-Hoon Choung

**Affiliations:** 1 Department of Otorhinolaryngology, Ajou University School of Medicine, Suwon 16499, Republic of Korea; 2 Department of Otorhinolaryngology-Head and Neck Surgery, Kangnam Sacred Heart Hospital, Hallym University College of Medicine, Chuncheon 24252, Republic of Korea

**Keywords:** hearing aid, HINT, HHIE, signal-to-noise ratio

## Abstract

(1) Background: For successful hearing aid (HA) use during daily life, an objective parameter reflecting the subjective satisfaction is required. We explored the aided hearing status, hearing in noise test (HINT) scores, and subjective outcomes to predict performance improvements in everyday living. (2) Methods: A total of 406 patients with hearing loss (HL) who were prescribed HAs were included and were divided into two groups according to the symmetricity of HL. The relationship between audiometric data and subjective questionnaires under unaided and aided (3 months) conditions were investigated. (3) Results: Patients with symmetric HL showed a significant HINT signal-to-noise ratio (SNR) change and significant increase in their subjective satisfaction questionnaire score under the bilateral HA condition. On the other hand, the HINT SNR change and subjective questionnaire score showed various significances according to the side of HA (better or worse hearing) in asymmetric HL HINT SNR and was significantly correlated with the subjective questionnaire score in symmetric HL patients and AHL patients with unilateral HA in their better ear. (4) Conclusions: The HINT SNR improvement after long-term HA use could be an effective tool for predicting the subjective satisfaction of HA use and HA validation.

## 1. Introduction

The number of patients suffering from hearing loss (HL) is increasing as the life expectancy increases. As HL becomes more severe, communication becomes more difficult, which reduces social activities and deepens feelings of isolation. In the report of The Global Burden of Disease Study, HL is the fourth leading cause of disability globally [1]. The progression of this hearing difficulty can cause several psychological problems and poor quality of life [2,3].

Hearing aids (HAs) are commonly recommended tools for non-surgical hearing rehabilitation. The prevalence of HA use was 14.2% in the United States during 1999–2006 [4]. The usage rate is quite low considering the high HL prevalence (20% to 40%) [4,5,6]. The reasons for poor regular HA usage are insufficient HA benefit, low motivation of the patient, and excessive expectation levels. Evaluating the benefit provided by HAs may be fundamental to achieving successful HA rehabilitation. There are two ways to assess the HA effectiveness: objective (aided threshold, speech discrimination score in aided condition, fitting status using the formula) and subjective (patient-reported outcome questionnaires). Subjective satisfaction is evaluated using questionnaires; hearing tests are conducted to optimize gain.

Even if improvement in objective HA assessment is confirmed through appropriate counseling and the fitting process, it often does not match the subjective dissatisfaction. This is because most objective evaluations are conducted in ideal environments (e.g., quiet and soundproof conditions) and focus on audibility. Patients with sensorineural HL have trouble in understanding speech in noisy environments, even when wearing HAs. Indeed, HAs are often not used and their purchase rate is low [5,7]. Users must be satisfied with their HAs during daily life. Therefore, an objective test that reflects the subjective satisfaction revealed by questionnaires is required.

Killion et al. performed a meta-analysis to evaluate the evidence of a good correlation between unaided pre-fitting speech measures and aided satisfaction on self-reported measures. Five studies were included in the review and there was no significant correlation between traditional unaided pre-fitting speech measures and aided satisfaction; only one study showed a correlation between a pre-fitting speech-in-noise test and self-reported satisfaction [8]. However, Killion et al. suggested that the use of pre-fitting signal-to-noise ratio (SNR) measures can provide an improved basis for providing realistic expectations during counseling and lead to the use of features that can bring more than half of HA wearers back to their normal ability to understand speech in noise [9].

In clinical settings, HL negatively affects speech understanding by a combination of attenuation and distortion factors. The attenuation factor corresponds to an increase in the threshold and the distortion factor is equivalent to a reduction in SNR. Killion proposed the concept of quantifying the increase in the distortion factor required for 50% correct recognition compared to a normal hearing person as ‘SNR loss’ [10].

The prediction of the everyday utility of HAs during an initial evaluation would greatly aid patient counseling. Walden investigated the relationship between various demographic and audiometric measures, and two measures of HA success in 50 HA wearers [11]. In that study, audiometric measures (audibility, suprathreshold distortion) were correlated with measures of HA success, and SNR loss was the best predictor of HA success in daily life. If the HA improved the ability to understand speech in noise, the difference between the unaided and aided SNR Quick Speech-in-Noise test (QuickSIN) score (the reduction in the SNR loss due to amplification) could predict success with amplification in everyday living. However, this predictive relationship might be attributed to the patient’s age.

For an objective test to be clinically useful, the data must be correlated with subjective satisfaction. Mendel investigated whether some newly developed speech recognition materials were sensitive enough to demonstrate objective HA benefit and whether such results would correlate well with patients’ subjective perceptions of that benefit [12]. In that study, the Revised Speech Perception in Noise test (R-SPIN), Hearing in Noise test (HINT), and QuickSIN were administered to 21 HA users. The difference between unaided and aided performance on these sentence tests and on the Hearing Aid Performance Inventory (HAPI) were compared. The R-SPIN, HINT Quiet threshold, and QuickSIN SNR loss were the most sensitive for assessing improvements in speech perception. The HAPI ratings rose when these three parameters improved.

Suprathreshold distortion effects of HL are the best predictors of everyday success with amplification. Therefore, a tool that can universally evaluate the degree of SNR loss is needed. The HINT is suitable for assessing functional hearing by adaptively measuring speech reception thresholds (SRTs) and has been used to evaluate HA performance [13]. In particular, scoring is based on the correct repetition of a sentence rather than a single word. Both ears are used to listen to sentences spoken in a quiet environment and in the context of noise from different directions. The HINT has been used to (1) evaluate the performance of HAs [14]; (2) evaluate performance of directional microphones in HAs [15,16]; (3) investigate the phenomenon of acclimatization to HAs [17]; (4) evaluate the issues related with cochlear implants [18,19]; and (5) evaluate functional hearing abilities required for hearing-critical jobs [20].

The Korean version of the HINT (K-HINT) has been developed and verified and is currently being used clinically. Here, we explored the aided hearing status and K-HINT scores and compared those with the subjective self-reported outcome to confirm performance improvements in the everyday living condition; we sought correlations between K-HINT scores and subjective satisfaction.

## 2. Materials and Methods

### 2.1. Reporting Guidelines

The manuscript was prepared in accordance with the STROBE guidance.

### 2.2. Participants

**Figure 1:**
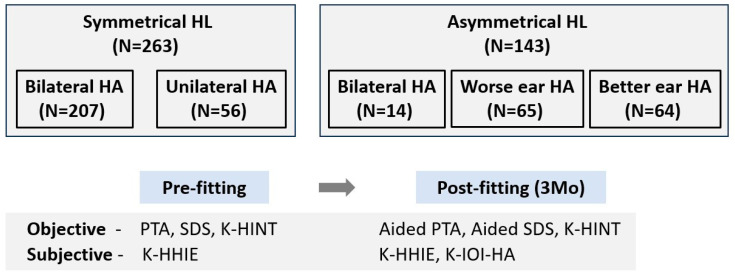
Flow chart of this study process. HL, hearing loss; HA, hearing aid; PTA, pure tone audiometry; SDS, speech discrimination score; K-HINT, Korean version of the Hearing in Noise Test; K-HHIE, Korean version of the Hearing Handicap Inventory for the Elderly; K-IOI-HA, Korean version of the International Outcome Inventory for Hearing Aids.

Of the patients who were prescribed HAs in the Department of Otolaryngology, Ajou University Hospital, Suwon, Republic of Korea, from September 2012 to December 2017, patients aged ≥ 10 years who continuously wore HAs for 3 months or whose functional gain test and K-HINT were completed at 3 months were enrolled (Figure 1). A total of 406 patients were included in this study. We calculated the required number of subjects using the G*Power program (version 3.1.9.6). Setting the parameters for matched pair analysis with an effect size of 0.5, α = 0.05, and power = 0.95, the necessary sample size was determined to be 54 individuals. Both the SHL group and AHL group exceeded the required sample numbers. Of the 406 patients, 211 (52%) were male and 195 (48%) were female; their mean age was 64.4 ± 15.4 years (10–19 years, 9; 20–29 years, 11; 30–39 years, 12; 40–49 years, 26; 50–59 years, 59; 60–69 years, 95; 70–79 years, 154; 80–89 years, 40). Patients were allocated to a symmetric HL (SHL) group (N = 263, 64.8%) and an asymmetric HL (AHL) group (N = 143, 35.2%).

### 2.3. Methods

SHL was defined as a mean threshold difference between ears of <10 dB; AHL was defined as a mean threshold difference of >20 dB. HA types were determined by otologists who evaluated hearing thresholds and speech discrimination scores (SDSs). In the SHL group, 207 patients wore bilateral HAs and 56 patients wore unilateral HAs. In the AHL group, 14 patients wore bilateral HAs, 65 patients wore an HA in their worse ear, and 64 patients wore an HA in their better ear. Receiver in the canal-type HAs (251 ears, 40%) were most commonly prescribed, followed (in order) by completely in the canal (213 ears 34%), in the canal (119 ears, 19%), behind the ear (38 ears, 6%), and invisible in canal types (6 ears, 1%). The manufacturers included Starkey (Eden Prairie, MN, USA), Resound (Copenhagen, Denmark), Widex (Copenhagen, Denmark), Siemens (Munich, Germany) and Phonak (Stäfa, Switzerland). Demographic and clinical data, diagnoses, and hearing/speech outcomes were retrospectively reviewed.

The routine fitting process is as follows. Unaided hearing using pure tone audiometry (PTA) and SDS was estimated. The PTA threshold was calculated as the mean of thresholds at 1000, 2000, and 4000 Hz. Based on audiological features and counseling, the appropriate HA was prescribed. Fine tuning procedures were repeated 1 and 3 months after HA use according to the patients’ feedback and audiological measurements. Aided SDSs were evaluated after 3 months of HA use; the functional gain test and K-HINT were also performed at that time. We analyzed the results of these audiometric evaluations in each group. The Korean version of the Hearing Handicap Inventory for the Elderly (K-HHIE) and the Korean version of the International Outcome Inventory for Hearing Aids (K-IOI-HA) were completed as self-reported outcome questionnaires to compare the extent of subjective hearing impairment before and after HA use and to measure patient satisfaction at the same time point. K-HHIE is designed to quantify the effects of hearing impairment on the emotional and social adjustment to HL in elderly people [21]. The K-HHIE, which consists of 25 questionnaires, is divided into two subscales: the emotional consequences of hearing impairment (13 items) and both social and situational effects (12 items). Each item is given a score of 0, 2, or 4, and the total score ranges from 0 to 100 with higher scores indicating a greater level of perceived handicap. K-IOI-HA is composed of seven different outcome domains to compare outcomes of HA fittings [22]: (1) the use of HAs, (2) the perceived benefits, (3) residual activity limitation, (4) satisfaction, (5) residual participation restriction, (6) the impact on others, and (7) change in quality of life. These domains are divided into the personal feelings of the HA wearer (1, 2, 4, 7) and interactions with others (3, 5, 6). The items are scored from 1 to 5, with higher scores indicating better results in the specific domain.

### 2.4. Statistical Analyses

Changes in SDS, K-HINT, and K-HHIE values were analyzed using the Wilcoxon signed-rank test or paired *t*-tests to compare unaided and aided data from each patient. Analyses of variance were utilized to compare K-IOI-HA scores. Pearson’s correlation analysis was used to analyze the correlation among parameters. All statistical analyses were performed with SPSS Statistics for Windows (version 21.0, IBM, Armonk, NY, USA). *p*-values < 0.05 were considered statistically significant.

## 3. Results

### 3.1. Audiometric Results and K-HHIE Scores

For all patients, the unaided mean pure tone thresholds and SDSs were 53.0 ± 10.3 dB HL and 56.3 ± 23.9%, respectively. The distribution of the mean threshold was as follows: >25 dB HL and ≤40 dB HL, 43 ears; >40 dB HL and ≤55 dB HL, 344 ears; >55 dB HL and ≤70 dB HL, 199 ears; >70 dB HL, 41 ears. The aided mean pure tone thresholds and SDSs values were 39.1 ± 7.8 dB HL and 67.7 ± 20.2%, respectively, which were significantly better than the unaided values (both *p* < 0.05). In terms of the K-HINT, the speech reception threshold in the quiet condition showed a significant decrease (unaided, 57.6 ± 17.7 dB; aided, 46.6 ± 14.2 dB, *p* < 0.05). Additionally, the SNR significantly decreased after HA use in noise from the front (unaided, 6.2 ± 8.9; aided, 4.8 ± 7.5, *p* < 0.05), right (unaided, 4.7 ± 10.0; aided, 3.5 ± 9.1, *p* < 0.05), left (unaided, 4.8 ± 9.4; aided, 3.2 ± 8.5, *p* < 0.05), and composite noise (unaided, 5.4 ± 8.9; aided, 4.1 ± 8.0, *p* < 0.05).

The overall K-HHIE score decreased from 42.2 ± 28.1 to 30.3 ± 24.6, the emotional subscale score decreased from 22.2 ± 15.1 to 16.0 ± 13.3, and the social subscale score decreased from 20.0 ± 13.8 to 14.5 ± 12.2 3 months after HA use (all *p* < 0.05).

### 3.2. The SHL Group

**Figure 2:**
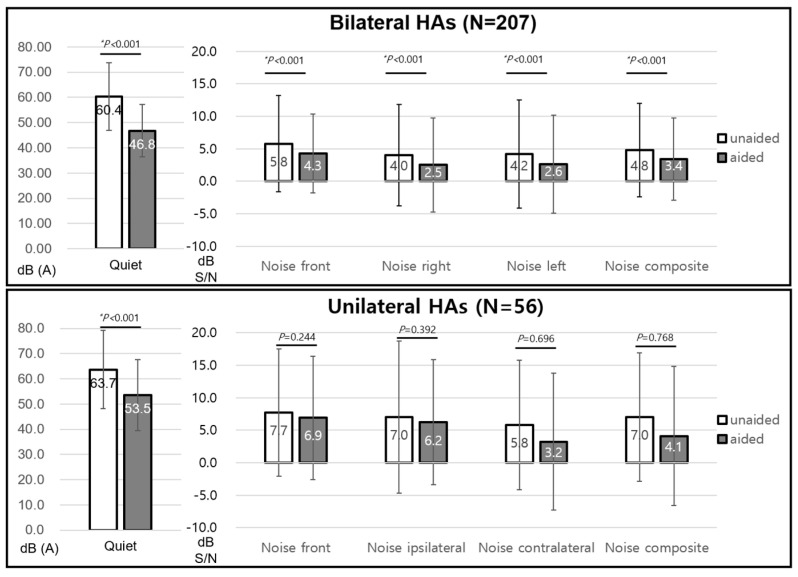
K-HINT in symmetrical hearing loss. K-HINT, Korean version of the Hearing in Noise Test; HA, hearing aid. * *P* < 0.05.

The unaided mean pure tone thresholds and SDSs were 51.3 ± 9.2 dB HL and 57.1 ± 23.2%, respectively. The aided values were 38.5 ± 7.9 dB HL and 67.8 ± 20.0%, respectively, which showed significant improvement (both *p* < 0.05). In the case of the K-HINT, the speech reception threshold in the quiet condition showed a significant decrease in patients with unilateral HA (unaided, 58.3 ± 12.3 dB; aided, 51.0 ± 9.3 dB, *p* < 0.05) and bilateral HAs (unaided, 60.0 ± 13.3 dB; aided, 46.0 ± 9.0 dB, *p* < 0.05). The SNR only significantly decreased in patients with bilateral HAs in noise from the front (unaided, 5.8 ± 7.4; aided, 4.3 ± 6.1, *p* < 0.05), right (unaided, 4.0 ± 7.8; aided, 2.5 ± 7.2, *p* < 0.05), left (unaided, 4.3 ± 8.3; aided, 2.6 ± 7.6, *p* < 0.05), and composite noise (unaided, 4.8 ± 7.2; aided, 3.4 ± 6.3, *p* < 0.05). However, the SNR change in patients with unilateral HA was not significant in all noise conditions (Figure 2).

**Figure 3:**
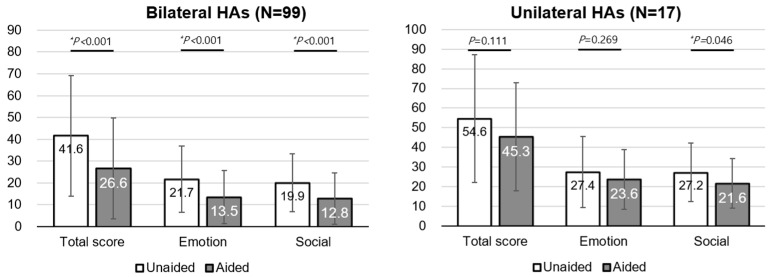
K-HHIE in symmetrical hearing loss. K-HHIE, Korean version of the Hearing Handicap Inventory for the Elderly; HA, hearing aid. * *P* < 0.05.

The K-HHIE in patients with bilateral HAs significantly decreased in the total score (unaided, 41.6 ± 27.7; aided, 26.6 ± 23.2, *p* < 0.05), the emotional subscore (unaided, 21.7 ± 15.2; aided, 13.5 ± 12.1, *p* < 0.05), and social subscore (unaided, 19.9 ± 13.3; aided, 12.8 ± 11.7, *p* < 0.05). However, only the social subscore significantly decreased in patients with unilateral HA (unaided, 27.2 ± 15.0; aided, 21.6 ± 12.8, *p* < 0.05) (Figure 3).

The K-IOI-HA scores did not differ between patients with bilateral (23.6 ± 4.1) and unilateral HAs (22.5 ± 4.1, *p* = 0.172). In detail, the personal feelings of the HA wearer (1, 2, 4, 7) and interactions with others (3, 5, 6) did not differ in their responses.

For patients with bilateral HAs, the K-HINT SNR was significantly positively correlated with the aided K-HHIE score (frontal noise, R = 0.303, *p* = 0.002; right noise, R = 0.390, *p* < 0.001; left noise, R = 0.284, *p* = 0.004; composite noise condition, R = 0.37, *p* < 0.001), but the aided threshold and aided SDS did not show any correlation with the aided K-HHIE score. On the other hand, the K-HHIE score difference value (aided value-unaided value) was not correlated with the K-HINT SNR, aided threshold, or aided SDS.

In the case of patients with unilateral HA, the K-HINT SNR was also significantly positively correlated with the aided K-HHIE score (frontal noise, R = 0.681, *p* = 0.002; ipsilateral noise, R = 0.566, *p* = 0.014; contralateral noise, R = 0.586, *p* = 0.011; composite noise condition, R = 0.682, *p* = 0.002), but the aided threshold and aided SDS did not show any correlation with the aided K-HHIE score. Interestingly, the K-HHIE score difference value was positively correlated with only the K-HINT SNR (noise front, R = 0.492, *p* = 0.038; noise ipsilateral, R = 0.481, *p* = 0.043; noise contralateral, R = 0.608, *p* = 0.007).

### 3.3. The AHL Group

**Figure 4:**
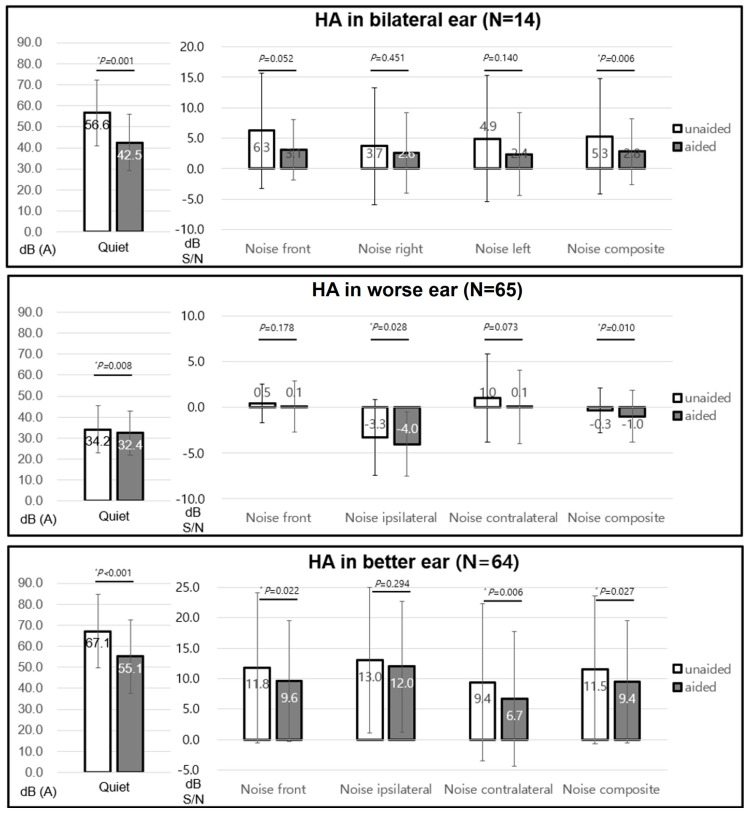
K-HINT in asymmetrical hearing loss. K-HINT, Korean version of the Hearing in Noise Test; HA, hearing aid. * *P* < 0.05.

The unaided mean pure tone thresholds and SDSs were 55.8 ± 11.5 dB HL and 54.8 ± 25.2%, respectively. The aided values were 40.1 ± 7.7 dB HL and 67.6 ± 20.5%, respectively, which showed significant improvement (both *p* < 0.05). For the K-HINT analysis, the AHL group was divided into patients with bilateral HAs, patients with HA in their worse ear, and patients with HA in their better ear. The speech reception threshold in the quiet significantly decreased in patients with bilateral HAs (unaided, 56.6 ± 15.6 dB; aided, 42.5 ± 13.4 dB, *p* < 0.05), HA in their worse ear (unaided, 34.2 ± 11.3 dB; aided, 32.4 ± 10.5 dB, *p* < 0.05), and HA in their better ear (unaided, 67.1 ± 17.6 dB; aided, 55.1 ± 17.6 dB, *p* < 0.05). Patients with bilateral HAs exhibited significant changes in SNR for frontal noise (unaided, 6.3 ± 9.5; aided, 3.1 ± 4.9, *p* = 0.052) and composite noise (unaided, 5.3 ± 9.5; aided, 2.8 ± 5.4, *p* < 0.05). In the case of unilateral HA, SNR significantly decreased in patients with HA in their worse ear for both ipsilateral noise (unaided, −3.3 ± 4.1; aided, −4.0 ± 3.5, *p* < 0.05) and composite noise (unaided, −0.3 ± 2.4; aided, −1.0 ± 2.8, *p* < 0.05) and in patients with HA in their better ear for frontal noise (unaided, 11.8 ± 12.3; aided, 9.6 ± 9.9, *p* < 0.05), contralateral noise (unaided, 9.4 ± 12.9; aided, 6.7 ± 11.0, *p* < 0.05), and composite noise (unaided, 11.5 ± 12.1; aided, 9.4 ± 10.0, *p* < 0.05) (Figure 4).

**Figure 5:**
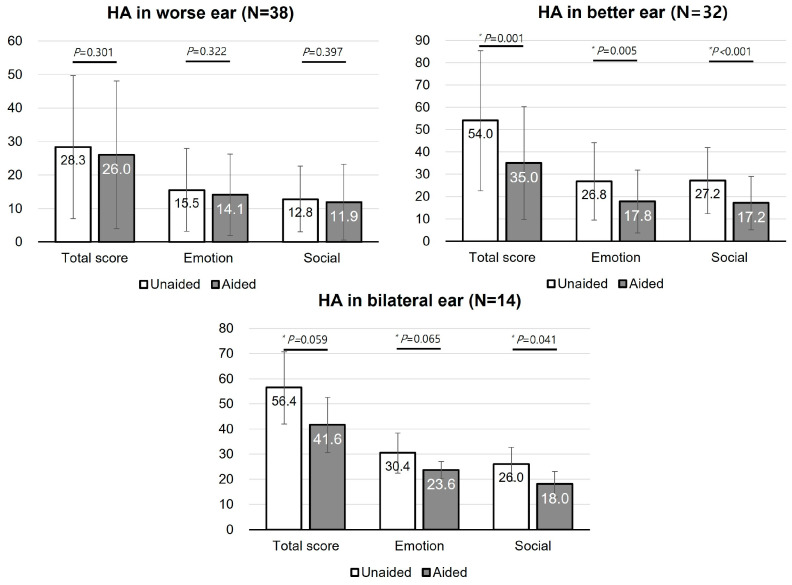
K-HHIE in symmetrical hearing loss. K-HHIE, Korean version of the Hearing Handicap Inventory for Elderly; HA, hearing aid. * *P* < 0.05.

The K-HHIE scores were distributed in various ways depending on the HA conditions. The patients with unilateral HA in their better ear showed a significant decrease in the K-HHIE in the total score (unaided, 54.0 ± 31.4; aided, 35.0 ± 25.3, *p* < 0.05), emotional subscore (unaided, 26.8 ± 17.3; aided, 17.8 ± 14.0, *p* < 0.05), and social subscore (unaided, 27.2 ± 14.8; aided, 17.2 ± 12.0, *p* < 0.05). Similarly, the K-HHIE in patients with bilateral HAs decreased in the total score (unaided, 56.4 ± 14.4; aided, 41.6 ± 11.0, *p* = 0.059), emotional subscore (unaided, 30.4 ± 8.0; aided, 23.6 ± 3.4, *p* = 0.065), and social subscore (unaided, 26.0 ± 6.7; aided, 18.0 ± 5.1, *p* < 0.05). On the other hand, the change in K-HHIE in patients with unilateral HA in their worse ear was not significant (Figure 5).

The K-IOI-HA scores did not differ among patients with bilateral HAs, unilateral HA in their better ear, or unilateral HA in their worse ear (*p* = 0.623). There were no significant differences between patients on any K-IOI-HA questionnaire subscale.

For patients with unilateral HA in their worse ear, the K-HINT SNR and aided threshold were not correlated with the aided K-HHIE score. Additionally, the K-HHIE score difference value was not correlated with the K-HINT SNR, aided threshold, or aided SDS. Patients with bilateral HAs were not analyzed due to the very small sample size.

In the case of patients with unilateral HA in their better ear, the K-HINT SNR and aided threshold were not correlated with the aided K-HHIE score. However, the K-HHIE score difference value was negatively correlated with the K-HINT SNR (frontal noise, R = −0.371, *p* = 0.034; ipsilateral noise, R = −0.474, *p* = 0.005; contralateral noise, R = −0.387, *p* = 0.026; composite noise, R = −0.411, *p* = 0.017) and positively correlated with the aided SDS score (R = 0.401, *p* = 0.021).

### 3.4. Other Predictive Factors for Subjective Satisfaction

The aided K-HINT SNR significantly increased and the aided SDS decreased with an increase in age in unilateral and bilateral HA conditions of the SHL group. However, the K-HHIE score difference value showed a tendency of negative correlation with age in patients with only bilateral HAs (R = −0.191, *p* = 0.057). In the AHL group, on the contrary, the aided K-HINT SNR significantly increased with age in patients with a unilateral HA condition and aided SDS significantly decreased with an increase in age in unilateral HA in the better ear. The K-HHIE score difference value showed no correlation with age.

The mean duration of HL was 5.3 ± 8.2 years (range, 0.5–70 years). The duration of HL did not show any correlation with the aided K-HINT SNR, aided threshold or aided SDSs in the SHL group and patients with unilateral HA in their worse ear in the AHL group; a significant correlation was found only with the contralateral noise condition in the aided K-HINT SNR (R = 0.324, *p* = 0.034) and aided SDS (R = −0.303, *p* = 0.051). Of note are the unaided K-HHIE scores of patients with unilateral HA in the SHL group (R = 0.593, *p* = 0.025).

The unaided threshold showed a significant correlation with the unaided K-HHIE score (R = 0.327, *p* < 0.001) and the K-HHIE score difference value (R = −0.315, *p* = 0.001) in patients with bilateral HAs in the SHL group. Similarly, the unaided threshold showed a significant correlation with the unaided K-HHIE score (R = 0.577, *p* = 0.008) in patients with unilateral HAs in the SHL group. In the case of the AHL group, the unaided threshold and SDS showed a significant correlation with the unaided K-HHIE score (R = 0.456, *p* = 0.008; R = −0.461, *p* = 0.007) and the K-HHIE score difference value (R = −0.375, *p* = 0.031; R = 0.481, *p* = 0.005).

## 4. Discussion

### 4.1. The SHL Group

The SHL group exhibited significant changes in aided thresholds, K-HINT score, and K-HHIE score only when HAs were worn in both ears. These could be explained by the advantages of binaural hearing such as improvement in speech recognition in noisy conditions as well as sound localization [23].

The K-HINT assesses difficulties in understanding everyday speech by exposing individuals to different SNRs. In patients with bilateral HAs, the aided threshold K-HINT SNR score significantly improved and the subjective K-HHIE scores also significantly improved. On the other hand, improvement in the K-HINT SNR score and K-HHIE were not significant in patients with unilateral HA, but the aided threshold significantly improved. In that sense, the K-HINT SNR score could reflect subjective satisfaction more directly than the aided threshold in SHL.

### 4.2. The AHL Group

In the AHL group, the K-HHIE exhibited greater improvement among patients with an HA in their better ear than among patients with an HA in their worse ear. The AHL patients with unilateral HA exhibited certain unique relationships between the audiological test and questionnaire data. For HA in the better ear condition, the changes in K-HINT SNR were significant under frontal, contralateral and composite noise conditions. In contrast, for HA in the worse ear condition, the change in K-HINT SNR was significant under ipsilateral noise conditions (ipsilateral noise and noise composite). However, the K-HHIE score significantly decreased only in patients with an HA in their better ear. Although the aided threshold decreased and the K-HINT SNR decreased in patients with HA in the worse ear under the ipsilateral noise condition, the effect of HA use on subjective satisfaction was not definite. An HA in the better ear did provide improvement in subjective satisfaction; the K-HINT SNRs when noise originated from the front and contralateral (worse hearing) sides significantly decreased, thereby significantly improving subjective satisfaction as revealed by changes in the K-HHIE score.

In the case of patients with bilateral HA, the relationship between the K-HINT SNR and K-HHIE showed a similar tendency to that of patients with unilateral HA in the better ear. This is because, as described previously, the effect of HA in the worse ear on subjective satisfaction is relatively trivial.

When changes in the K-HINT were compared between the SHL and AHL groups, changes among AHL patients more clearly reflected the effects of HAs.

### 4.3. Predictive Variable for Successful HA Adaptation

As expected, we found that the aided thresholds significantly decreased after HA use in patients with both SHL and AHL. However, the change in K-HINT score differed according to the HL extent, HA condition, and noise location. In that sense, which is the more appropriate factor reflecting subjective satisfaction between the aided threshold and K-HINT SNR should be considered. Theoretically, the aided thresholds to pure tone stimuli in the soundproof booth are useful for estimating the general amplification provided by HAs, but this quiet condition could be different from real life. Therefore, speech reception measured under the various noisy conditions encountered in everyday life is appropriate for evaluating the subjective HA effect. The SNR reduction, not aided threshold improvement, is suitable for evaluation of HA effectiveness, supporting the findings of previous studies that focused on changes in SNR after HA use [11,12].

### 4.4. Audibility and SNR Loss

Kochkin reported that only 59 patients were satisfied with HA performance among 3000 HA users [24]. Many studies have searched for the factors predicting successful HA use. Plomp suggested that the effect of HL on speech understanding could be explained by a combination of attenuation and distortion factors [25]. The attenuation factor simply means the loss of threshold sensitivity (audibility). This audibility is the most fundamental hearing need in patients with HL, and it could be overcome via amplification. In this study, aided thresholds of SHL and AHL groups also significantly decreased. However, Walden reported that audibility measures were unrelated to outcome measures [11], which was supported by this study. This irrelevance between the audibility measures and the HA success means that measures of audibility did not predict everyday success with amplification. The distortion factor is equivalent to an SNR reduction irrespective of amplification. This distortion factor is affected by central auditory processing and cognitive deficits, as well as peripheral influences, which is not compensated by conventional amplification [11]. Killion suggested that the distortion factor may be quantified by the “SNR loss”, defined as the increase in SNR required for a person with impaired hearing to achieve 50 percent correct recognition compared to a person with normal hearing [10]. In this current study, the K-HINT SNR also shows a meaningful relationship with K-HHIE rather than the aided threshold. In the case of the K-HINT, the test is conducted with different noise directions: front, left, and right, making it possible to accurately evaluate SNR according to the degree of hearing loss, symmetry, and HA wearing condition. Changes in the K-HINT may not be useful in certain types of HL patients with or without an HA or with bilateral HAs; however, compared with the aided threshold, changes in the K-HINT more objectively measure the effect of HA use.

Age is considered another factor predicting successful HA rehabilitation. A large-scale study by Hosford-Dunn and Halpern was performed to investigate the relationship between patient-related variables such as age, gender, years of HA use, pure tone average and global satisfaction scores for successful HA fitting [26]. In that study, age was only negatively correlated with satisfaction scores. Previous studies revealed that suprathreshold auditory processing abilities diminish with age, including frequency and intensity discrimination and temporal processing [27,28,29]. In this regard, the distortion factor becomes prominent with age. In this study, although the age of 249 patients (61.3%) ranged from 60 to 79 years, a similar tendency of the effect of age on the K-HINT SNR was identified.

### 4.5. Self-Reported Questionnaires

The K-HHIE self-assessment evaluates the hearing disability on emotional and social adjustment in older adults [21]. Therefore, the comparison of K-HHIE scores between the unaided and long-term aided conditions could be useful for subjective satisfaction for wearing HA. On the contrary, the K-IOI-HA is only focused on assessing HA effects and not hearing disability [30]. The K-IOI-HA score indicates subjective benefits derived from HAs; higher scores indicate better HA outcomes [31]. However, since the K-IOI-HA evaluation is generally conducted after wearing the HA for two weeks, it is not necessarily appropriate for the estimation of long-term evaluation. Additionally, given that the number of questions is small and the content is simple, it is difficult to evaluate the effectiveness of HA in each listening situation or specific and diverse satisfaction levels after wearing an HA. Additionally, in this study, the K-HHIE scores significantly improved in SHL patients with bilateral HAs and in AHL patients with HAs in either both ears or the better ear. However, the K-IOI-HA scores did not significantly differ between the SHL and AHL groups. The K-HHIE is a more beneficial tool than the K-IOI-HA for long-term analysis of successful HA adaptation.

## 5. Conclusions

The extent of K-HINT score improvement after long-term HA use could be an effective marker for predicting the subjective satisfaction of HA use and HA validation along with other currently used auditory evaluation tests. In addition to K-HINT, various test methods quantifying the distortion effects should be supplemented. Developing a protocol that can interpret the K-HINT SNR and apply it to a fitting procedure according to the type and extent of HL, age, and HA wearing condition may be helpful for successful HA rehabilitation.

## Data Availability

The datasets generated and/or analyzed in the current study are available from the corresponding author upon reasonable request.
